# Professional Resistance: Why Korean Medical Students are Boycotting Over Increasing Medical School Places

**DOI:** 10.5334/pme.1426

**Published:** 2024-11-27

**Authors:** Anna de Beer, Adelina S. Werner, Seunggeun Kim, Frederike A. Jenne

**Affiliations:** 1 School of Medicine, University of St Andrews, Scotland, United Kingdom; 2 Barts and the London School of Medicine and Dentistry, Queen Mary University of London, England, United Kingdom; 3 School of Medicine, University Hospital of Regensburg, Regensburg, Germany; 4 School of Medicine, Catholic University of Korea, Seoul, South Korea

## Abstract

In February 2024, medical students in South Korea began submitting leave-of-absence requests in protest of a 65% increase in the number of medical school places in 2025. 14,000 medical students have boycotted classes and 12,000 doctors have resigned en masse. The 2024 Korean medical student collective action highlights the ‘power’ of medical students, the need for independent medical licensing bodies and the risks to the quality of medical education when places in medical schools are rapidly increased. The South Korean healthcare system is consistently ranked as one of the best in the world but there is now a significant risk that few new doctors will graduate and enter the workforce in March 2025.

## Introduction

The South Korean healthcare system is consistently ranked as one of the best in the world [1,2]. Despite this, criminal lawsuits in high-risk specialties, doctor shortages in essential medical specialties, and 80-hour work weeks are all part of a Korean doctor’s reality. In February 2024 the government announced a seemingly simple solution to some of these issues – increase the number of medical school places by 65%. This proposal however, brought the already brewing medical crisis to a head. Almost 14,000 medical students have been boycotting university and 12,000 doctors resigned en masse in protest against the government’s proposal. As of October 2024, the South Korean medical crisis is ongoing, with no resolution in sight.

Two sides can be identified in this conflict: the Korean government and the doctors/ medical students actively protesting the change. The administration of President Yun Suk Yeol argues that the ageing population combined with the low number of doctors per capita compared to other OECD countries [3], will result in a deficit of 15,000 doctors by 2035 [4]. The evidence cited however, is unclear [5]. Doctors and medical students show frustration, claiming this reduces a host of deeper problems to a single quantitative issue, and that Korea does in fact have enough doctors [6]. The mass resignations and university boycotts are acts of professional resistance that protest a systemic pattern of doctor exploitation and threats to their professional autonomy, that ultimately affect the quality of patient care.

To gain a deeper understanding of the broader academic context, we discuss the concept of professional resistance in healthcare, before applying these principles to the Korean situation. Finally, we discuss the impact/ consequences of these acts of professional resistance, and how the current conflict may be resolved.

We conducted seven informal unstructured interviews with medical students, doctors and university professors to further our insight. Per our academic institution guidelines (University Hospital of Regensburg), ethical approval was not required for these interviews as none of the criteria for requiring ethical review were met. All quotes and identifying information are presented with explicit consent by the interviewees.

## Professional Resistance in Healthcare

To date, literature on physician professional resistance remains rare, especially outside the USA [7]. Ellaway and Wyatt conceptualized professional resistance as “*expressions of condemnation of social harm and injustices, with the intent of stopping them, preventing them from recurring, and/or holding those responsible for them to account.”* [8] These acts, whether altruistically motivated or in self-advocacy, are a moral stance against perceived injustices, driven by the duties and commitments of one’s role [9].

Forms of resistance can be covert or overt. Wyatt et al. discuss the complexities of covert and overt resistance and how they interact, noting that everyday covert resistance, which has not been formally organised, aims to avoid detection [9]. This is not to say that these forms of resistance are any less political. Looking at the 2024 professional resistance in South Korea offers a tangible illustration of these conceptual principles.

## Professional Resistance in South Korea in 2024

The 2024 mass resignations and university boycotts are a symptom of a deep distrust in the government’s will and ability to address issues raised by healthcare professionals. Korean doctors’ professional resistance can be classified as an overt opposition to governmental policies in an act of self-advocacy. As Korean doctors are prohibited from forming unions and striking, their resistance took the form of 90% of resident doctors resigning [10]. Medical students turned to more covert forms of protest; they handed in university leaves-of-absences, with many male students choosing this time to complete their mandatory military service [11]. As of July 22^nd^, only 5.3% of all fourth-year medical students are registered for the 2025 Korean Medical Licensing exam, with only 2.7% of students across all year groups attending classes [12]. This results in students being held back by a year, clogging the system and thus keeping the number of new doctors graduating low.

Many medical students are using the time-off from university to discreetly prepare for international medical licensing exams with the intent to emigrate abroad. Doctors and students ‘voting with their feet’ – making their grievances clear in a system where they feel disempowered by exercising autonomy over their very presence – devastates a medical system as much as it makes a political statement [13]. A cross-sectional study post-policy announcement revealed that 41.3% (p < 0.001) of medical students wish to train abroad – mostly in the USA [14]. The prospective exodus of talent and workforce poses a significant risk to the Korean healthcare system.

## Why Students are Boycotting

The increase in medical school places was merely the catalyst for the collective action, with many of the reasons for the protest lying beyond medical school admissions. The protesters’ primary motivations fall into four main categories: risk of criminal prosecution, working conditions, threats to professional autonomy and quality of education.

Whilst doctors and the government disagree on the existence of an absolute lack of doctors, they agree on the relative lack of doctors in ‘essential’ specialties such as emergency medicine, obstetrics and pediatrics. Korean medical students and doctors avoid these ‘essential’ specialties, which typically involve more high-risk procedures, due to the increased risk of criminal prosecution. In most countries, medical lawsuits are routinely handled under civil law. By contrast, in Korea, an average of 754.8 doctors per year are prosecuted under criminal charges. Compared to 17.2 doctors per year in Germany and 51.5 in Japan [15]. Korean medical students are calling for greater professional protection under the law.

In addition to the threat of criminal prosecution, doctors’ sub-ideal working conditions are shaped by excessive work hours and load. The Korean healthcare system is universal, providing medical coverage through a single-payer National Health Insurance System. The fixed fees-for-services are often lower than the actual costs incurred by healthcare providers [16]. This pricing structure forces doctors to see a large number of patients, often seeing 80 patients a day in outpatient clinics. The high patient volume increases the risk of burnout and compromises the quality of patient care. The imposition of low fee-for-service rates is perceived by many doctors/students as an assault on their professional autonomy. They argue that the state’s control over healthcare pricing restricts their ability to make clinical decisions based solely on patient needs, pushing volume over quality. There are further concerns that an increase in doctors could heighten market competition, driving down salaries as patients spread out over more providers, potentially incentivising doctors to over-treat patients. Hence adding more physicians to the workforce may not alleviate the high workload.

In many countries, medical licenses are regulated by independent licensing bodies (i.e. the General Medical Council in the UK). In Korea however, the Ministry of Health, a government entity, is responsible for regulating medical licenses. This system strips away professional autonomy as the government holds the power to suspend or entirely revoke medical licenses when disputes arise. When 90% of resident doctors resigned this year [10], the government threatened to suspend doctors’ medical licenses if the doctors did not return to work immediately [17]. As a result of these abuses of employee rights and autonomy, the medical community is seeking greater professional jurisdiction, advocating for more regulatory independence.

Lastly, expanding medical school places by 65% with only a year’s notice poses a risk of dilution of education quality. When asked about his reasons for joining the boycott, final-year medical student CS explained: ‘*There needs to be a balance between availability of hospital clinical teaching and the number of medical students […] When you add in 2,000 additional medical students, you break this balance and overwhelm the system*’(25/04/2024). High-quality medical education relies not only on lecture-based teaching but also on clinical skills, hospital teaching and small-scale discussion-based teaching, which may be compromised with a larger student cohort.

The reasons underlying the resistance are numerous and extend beyond the opposition to increasing medical school places. Many of the grievances are structurally entrenched in the healthcare system, but all motivations driving the current protests revolve around protecting the ability of doctors to provide high-quality care and maintain their professional autonomy.

## The Consequences

The ongoing (as of the 15^th^ of October 2024) professional resistance has created significant disruptions in the South Korean healthcare system. The immediate impact on healthcare delivery is primarily due to the resignation of nearly all resident doctors, further compounded by some senior doctors resigning in solidarity. The chairperson of the Korean Emergency Medicine Association, Lee Hyung-min noted to the media that, ‘*The capacity of hospitals is less than half of what it was before the collective action, with the utilization rate of major hospital beds starting to fall below 50 per cent.*’ [18] In hospitals, mainly senior doctors remain to uphold a minimum of patient care, the remaining staff are consequently burdened with increased workloads. A professor of internal medicine told the Hankyoreh newspaper, ‘*some professors are quitting their job, not to join the collective action, but because they are experiencing burnout.*’ [18] In an attempt to mitigate these problems, the government deployed military doctors and extended the hours of public hospitals. These measures however, are insufficient temporary fixes that fail to address the underlying dilemma.

The long term impact and state-level responsiveness will likely be due to medical students’ acts of professional resistance. University hospitals in South Korea are heavily reliant on lower-paid resident doctors [19], but with the majority resigned, and few new doctors due to graduate, hospitals are having to restructure their medical provision based on decreased available staffing. Several university hospitals are facing the threat of imminent bankruptcy [20]. The past eight months have shown that if medical systems are reliant on resident doctors, even first-year students can hold great influence over the long-term delivery of healthcare, as when these students boycott, they will starve hospitals of a resident workforce five years down the line.

In light of the risk of ‘brain drain’ as students/doctors seek to move abroad, in March 2024, the government threatened to administratively prevent resigned doctors from obtaining US medical licenses by making it impossible to obtain the necessary visas [21]. The attempt to limit doctors’ free movement foregrounds the uniquely challenging environment in which professional resistance is taking place in South Korea, as such acts of resistance must change and develop as the system adjusts and evolves to contain it [9]. For many, the state’s interventionist approach represents an erosion of professional freedom. As a result, the standoff continues, fuelled by the medical community’s insistence on their right to practice medicine with autonomy, free from excessive state interference and unrealistic economic constraints. The challenge now lies in bridging the divide between both sides.

## Finding a Resolution

The government and medical community both acknowledge the social harm to the larger community owing to the boycott and underlying issues within the healthcare system. They diverge however in opinion as to the nature of the problem and the route to a solution. Resolving the current impasse requires dialogue and collaborative problem-solving [22]. Both sides must engage to co-create solutions that address the need for increased healthcare capacity alongside concerns over education quality, the medico-legal judicial system and professional autonomy.

The mass professional resistance places the government in a difficult situation. The irony is that the government policy to increase medical school places and consequently the number of doctors has instead resulted in South Korea facing the most acute doctor shortage in recent history.

The primary aim should be to work towards a timely resolution that sees medical education resumed. It is now unfeasible for final-year students to graduate as expected in March 2025, yet every effort must be made to ensure that the boycott does not continue. In line with the four pillars of principled negotiation, mutual antagonism needs to be put aside to enable constructive solutions [22]. Common interests may be found in increasing the number of doctors in ‘essential’ specialties, perhaps through financial incentives and policies to protect doctors from disproportionate criminal prosecution. Policies aimed at improving working conditions, enhancing both patient and doctor safety, could also serve as agreeable common ground for both parties in reaching a resolution to the crisis. For a lasting solution, all parties should adopt a long-term approach that focuses on building trust by facilitating greater professional autonomy – establishing an independent medical licensing body could be an important step towards rebuilding this trust.

## Conclusion

The 2024 Korean medical student and doctor protests highlight the critical role of professional resistance in shaping healthcare policy. The mass boycotts and resignations have brought to light significant problems within South Korea’s healthcare system, including the lack of professional autonomy, low fee-for-service rates, and concerns over the quality of future medical education. While the government’s intention to increase the number of medical school places was aimed at addressing projected doctor shortages, it has inadvertently triggered widespread unrest among the medical community. The new policy, seen as a top-down approach that neglects the voices of healthcare professionals, has been met with strong opposition, underscoring the need for collaborative policymaking. The power of professional resistance in this context has demonstrated the significant influence that doctors and students can exert on shaping healthcare policy, evidencing that collective action can compel governments to reconsider healthcare reforms.

The South Korean experience offers valuable insights for other nations facing similar challenges, offering a reminder that sustainable healthcare policy must balance the interests of all stakeholders. As the government and medical community navigate the current tensions, it is crucial to seek collaborative solutions that honour professional autonomy and address public health needs.

## References

[1] South Korea. Legatum Prosperity Index 2023 [online]. https://www.prosperity.com/globe/south-korea (accessed 02 Sept 2024).

[2] The 2021 Global Health Security Index [online]. GHS Index; 2021. https://ghsindex.org/ (accessed 02 Sept 2024).

[3] Organisation for Economic Co-operation and Development. Practising doctors per 1 000 population, 2011 and 2021 (or nearest year). Health at a Glance 2023: OECD Indicators. OECD Publishing; 2023. https://www.oecd-ilibrary.org/sites/9f24c36f-en/index.html?itemId=/content/component/9f24c36f-en#figure-d1e31163-f5d024543f.

[4] Ministry of Health and Welfare. Press Release. 02 Feb 2024. https://www.mohw.go.kr/board.es?mid=a20401000000&bid=0032&list_no=1480151&act=view.

[5] Han S. Three reports providing evidence for ‘increasing the number of medical school students by 2,000’… What do the authors think? KBS News [online]. 21 Feb 2024. https://news.kbs.co.kr/news/pc/view/view.do?ncd=7895654 (accessed 02 Sept 2024).

[6] Korean Medical Students Association (@kmsaoffical). International Statement. Instagram photo. 03 March 2024. https://www.instagram.com/p/C4DlCOcPEp3/?img_index=1 (accessed 25 April 2024).

[7] Wyatt TR, Ma TL, Ellaway RH. Physician resistance to injustice: A scoping review. Soc Sci Med. 2023; 320: 115727. DOI: 10.1016/j.socscimed.2023.115727 36736054

[8] Ellaway RH, Wyatt TR. What Role Should Resistance Play in Training Health Professionals? Acad Med. 2021; 96(11): 1524–8. DOI: 10.1097/ACM.0000000000004225 34232150

[9] Wyatt TR, Jain V, Ma TL. ‘When I stood up for something it’s because I felt a… moral violation’: Trainees’ acts of resistance against social harm and injustice. Med Educ. 2024; 58(4): 457–63. DOI: 10.1111/medu.15256 37975514

[10] Cho HY. 전공의 집단 사직 일주일…환자 ·의료진 “모두 힘들어 [One week after residents’ mass resignation… patients and healthcare professionals: “All having challenges”]. KBS news [online]. 26 Feb 2024. https://n.news.naver.com/article/056/0011669634?sid=102 (accessed 05 Sept 2024).

[11] Song S. Medical students on leave of absence join army instead of returning to school. Korea Biomedical Review [online], 04 April 2024. https://www.koreabiomed.com/news/articleView.html?idxno=25046 (accessed 06 Sept 2024)

[12] Song S. Medical students threaten to extend class boycott into 2nd semester. Korea Biomedical Review [online], 06 Aug 2024. https://www.koreabiomed.com/news/articleView.html?idxno=24767#:~:text=The%20second%20semester%20will%20soon,attend%20classes%20simultaneously%20next%20year (accessed 01 Sept 2024).

[13] Rao KD, Mehta A, Noonan C, Peters MA, Perry H. Voting with their feet: Primary care provider choice and its implications for public sector primary care services in India. Soc Sci Med. 2024; 340: 116414. DOI: 10.1016/j.socscimed.2023.116414 38039764 PMC10828545

[14] Kim GH, Park JE, Yeon JP, Lee JS. The Impact of Korea’s Essential Medicine Policy Package on Medical Students’ Career Intentions: A Cross-Sectional Study. Korean Medical Education Congress; 24 May 2024; Seoul. p339

[15] Research Institute for Healthcare Policy of Korean Medical Association. Current Status and Implications of Penalization of Medical Practice. 09 Oct 2022. https://rihp.re.kr/bbs/board.php?bo_table=research_report&wr_id=338.

[16] Kim Y, Kim NE, Kim YH, et al. 신포괄수가제 모형 개선 및 의료 질 관리 방안 연구 [Study on improving the new comprehensive payment model and medical quality management strategies]. Health Insurance Review and Assessment Service [online]. Jan 2020. https://repository.hira.or.kr/handle/2019.oak/2272 (accessed 02 Sept 2024).

[17] Kim Y. Korea begins process to suspend medical licenses of over 7,000 protesting trainee doctors. Hankyoreh [online]. 05 March 2024. https://english.hani.co.kr/arti/english_edition/e_national/1131005.html (accessed 02 Sept 2024).

[18] Kim Y, Lim J, Cheon H. After 2 months of delayed, denied medical care, Koreans worry worst may be yet to come. Hankyoreh [online]. 19 March 2024. https://english.hani.co.kr/arti/english_edition/e_national/1137366 (accessed 04 May 2024).

[19] Lee HJ. ‘빅5 병원’ 의사 중 전공의 40%… 기형적 의료체계가대란 불렀다 [Residents constitute 40% of doctors in the ‘big 5 hospitals’… abnormal healthcare system resulted in crisis]. Seoul Newspaper [online]. 22 Feb 2024. https://www.seoul.co.kr/news/society/health-medical/2024/02/22/20240222008005 (accessed 05 Sept 2024).

[20] Kwak S. National university hospitals face bankruptcy risk amid $912 mil. revenue drop. Biomedical Review [online]. 07 July 2024. https://www.koreabiomed.com/news/articleView.html?idxno=24483 (accessed 03 Sept 2024).

[21] Kwak S. Korea to block resigned trainee doctors from getting US medical license. Korea Biomedical Review [online]. 22 March 2024. https://www.koreabiomed.com/news/articleView.html?idxno=23637 (accessed 25 April 2024).

[22] Wyatt TR, Graves L, Ellaway RH. “Those Darn Kids”: Having Meaningful Conversations about Learner Resistance in Medical Education. Teaching and Learning in Medicine, 1–8. DOI: 10.1080/10401334.2024.2354454 38775111

